# *Notes from the Field:* Outbreak of Human Immunodeficiency Virus Infection Among Persons Who Inject Drugs — Cabell County, West Virginia, 2018–2019

**DOI:** 10.15585/mmwr.mm6916a2

**Published:** 2020-04-24

**Authors:** Amy Atkins, R. Paul McClung, Michael Kilkenny, Kyle Bernstein, Kara Willenburg, Anita Edwards, Sheryl Lyss, Erica Thomasson, Nivedha Panneer, Nathan Kirk, Meg Watson, Elizabeth Adkins, Elizabeth DiNenno, Vicki Hogan, Robyn Neblett Fanfair, Kathleen Napier, Alison D. Ridpath, Michelle Perdue, Mi Chen, Tamara Surtees, Senad Handanagic, Heather Wood, Daphne Kennebrew, Caitlin Cohn, Samira Sami, Scott Eubank, Nathan W. Furukawa, Bridget Rose, Antoine Thompson, Lauren Spadafora, Carolyn Wright, Shawn Balleydier, Dawn Broussard, Pam Reynolds, Neal Carnes, Nils Haynes, Tobey Sapiano, Shannon McBee, Ellsworth Campbell, Samantha Batdorf, Melissa Scott, Miracle Boltz, David Wills, Alexandra M. Oster

**Affiliations:** ^1^West Virginia Bureau for Public Health, West Virginia Department of Health and Human Resources; ^2^Division of HIV/AIDS Prevention, National Center for HIV/AIDS, Viral Hepatitis, STD, and TB Prevention, CDC; ^3^Cabell-Huntington Health Department, West Virginia; ^4^Division of STD Prevention, National Center for HIV/AIDS, Viral Hepatitis, STD, and TB Prevention, CDC; ^5^Marshall University Joan C. Edwards School of Medicine, Huntington, West Virginia; ^6^HIV/AIDS Bureau, Health Resources and Services Administration, U.S. Department of Health and Human Services, Washington, DC; ^7^Division of State and Local Readiness, Center for Preparedness and Response, CDC; ^8^Public Health Associate Program, CDC; ^9^Epidemic Intelligence Service, CDC.

In January 2019, West Virginia Bureau for Public Health (WVBPH) surveillance staff members noted an increase in diagnoses of human immunodeficiency virus (HIV) infection among persons who inject drugs in Cabell County, West Virginia (population approximately 91,900*). Cabell County, part of a medium-sized metropolitan statistical area and home to the city of Huntington (population approximately 46,000^†^), had historically high rates of substance use disorder but low rates of HIV infection (*1*). During 2013–2017, an annual average of two diagnoses of HIV infection had occurred among Cabell County persons who inject drugs; however, in 2018, 14 diagnoses occurred, including seven in the fourth quarter.

WVBPH requested assistance from CDC for a public health investigation and response. WVBPH, the Cabell-Huntington Health Department (CHHD), and CDC investigated to characterize the outbreak and guide public health interventions. Initial investigation found that at the time this increase in diagnoses of HIV infection was detected, access to HIV testing and preexposure prophylaxis (PrEP) in Cabell County was limited. Although a harm reduction program, including access to sterile syringes, had been operating at CHHD since September 2015, stricter requirements, including proof of Cabell County residency, were initiated in May 2018, which limited access to these services. Moreover, knowledge about HIV, the outbreak, and treatment for substance use disorder was low, and initiation of treatment for HIV or substance use disorder among persons who inject drugs was also low.

Interventions to address these challenges were rapidly scaled up by staff members from WVBPH, CHHD, CDC, and community partners. A case was defined as a new diagnosis of HIV infection during January 1, 2018–October 9, 2019 in 1) a person who injects drugs (regardless of other risk factors), who resided or was homeless in Cabell County at diagnosis, whose HIV diagnosis occurred in Cabell County, or who reported injecting drugs or accessing syringe services in Cabell County; or 2) a sex or needle-sharing partner of someone meeting criterion 1; or 3) a person whose HIV-1 polymerase sequence was linked at a genetic distance of ≤0.5% to that of a person meeting criterion 1 (*2*).

CDC staff members provided surge capacity to interview persons with a new diagnosis of HIV and offer HIV prevention services to approximately 600 identified partners and social contacts of these persons. Screening events were conducted to test persons at high risk, provide health education, and link or reengage persons in HIV care. The team worked with local hospitals, clinics, substance use disorder treatment providers, and community-based organizations to scale up HIV testing at locations where persons who inject drugs accessed services. A social network strategy driven by peer recruitment was implemented to reach persons who inject drugs who were not already engaged in the harm reduction program and their sexual and social contacts. The team also partnered with local infectious disease providers and support staff members to improve linkage to HIV and hepatitis C virus care and reengagement for persons who were no longer in care. Interviews were conducted with persons who inject drugs who also reported exchanging sex for money or drugs to identify barriers (e.g., stigma, discrimination, and location and hours of services) that might hinder access to prevention services and to guide service delivery. In addition, the team expanded access to PrEP by training new providers and supporting PrEP implementation at CHHD and two community health systems.

As of January 26, 2020, a total of 82 persons had met the case definition (Table). Among 61 (74%) persons with a CD4^+^ count measured ≤3 months after diagnosis, median CD4^+^ count was 583 (range = 6–1,057), indicating that many infections were recent. Among 50 persons who had an available HIV-1 polymerase sequence test result, 46 (92%) were part of a single cluster of closely related infections, indicating rapid transmission. As a result of the combined response activities, approximately 450 new clients enrolled in the harm reduction program, including approximately 50 persons living with HIV infection. CDC assisted in the development of educational campaigns and materials related to HIV infection, substance use disorder, stigma, PrEP, safe injection, and safe syringe and needle disposal for persons who inject drugs and community members. WVBPH and CHHD continue to work together in this response, and WVBPH is improving preparedness for detecting and responding to other clusters and outbreaks statewide through enhanced surveillance.

**TABLE:** Characteristics of persons with outbreak-associated human immunodeficiency virus infection — Cabell County, West Virginia, January 1, 2018–October 9, 2019*

Characteristic	No. (%)
**Total**	**82 (100)**
**Sex**	
Male	49 (60)
Female	33 (40)
**Age group (yrs)**	
<20	0 (0)
20–39	61 (74)
≥40	21 (26)
**Race/Ethnicity**	
White, non-Hispanic	75 (92)
Black, non-Hispanic	1 (1)
Hispanic	1 (1)
Other	5 (6)
**Transmission category**	
Injection drug use	75 (92)
Male-to-male sex and injection drug use	6 (7)
Male-to-male sex	1 (1)
**Exchanged sex for money or drugs**	24 (29)
**Laboratory evidence of current or past hepatitis C virus infection**	72 (88)

* Data were last updated on January 26, 2020.
